# Toxoplasma gondii-Induced Neutrophil Extracellular Traps Amplify the Innate and Adaptive Response

**DOI:** 10.1128/mBio.01307-21

**Published:** 2021-10-05

**Authors:** Farlen J. B. Miranda, Bruno C. Rocha, Milton C. A. Pereira, Larissa M. N. Pereira, Erikson H. M. de Souza, Ana P. Marino, Pedro A. C. Costa, Daniel V. Vasconcelos-Santos, Lis R. V. Antonelli, Ricardo T. Gazzinelli

**Affiliations:** a Instituto Rene Rachou, FIOCRUZ-MG, Belo Horizonte, Minas Gerais, Brazil; b Department of Medicine, University of Massachusetts Medical School, Worcester, Massachusetts, USA; c Departamento de Bioquímica e Imunologia, Universidade Federal de Minas Gerais, Belo Horizonte, Minas Gerais, Brazil; d Departamento de Oftalmologia e Otorrinolaringologia, Faculdade de Medicina da Universidade Federal de Minas Gerais, Belo Horizonte, Minas Gerais, Brazil; University of California, Irvine

**Keywords:** NET, *Toxoplasma gondii*, neutrophils

## Abstract

Toxoplasmosis affects one-third of the human population worldwide. Humans are accidental hosts and are infected after consumption of undercooked meat and water contaminated with Toxoplasma gondii cysts and oocysts, respectively. Neutrophils have been shown to participate in the control of T. gondii infection in mice through a variety of effector mechanisms, such as reactive oxygen species (ROS) and neutrophil extracellular trap (NET) formation. However, few studies have demonstrated the role of neutrophils in individuals naturally infected with T. gondii. In the current study, we evaluated the activation status of neutrophils in individuals with acute or chronic toxoplasmosis and determined the role of T. gondii-induced NET formation in the amplification of the innate and adaptive immune responses. We observed that neutrophils are highly activated during acute infection through increased expression of CD66b. Moreover, neutrophils from healthy donors (HDs) cocultured with tachyzoites produced ROS and formed NETs, with the latter being dependent on glycolysis, succinate dehydrogenase, gasdermin D, and neutrophil elastase. Furthermore, we observed elevated levels of the chemokines (CXC motif) CXCL8 and (CC motif) CCL4 ligands in plasma from patients with acute toxoplasmosis and production by neutrophils from HDs exposed to *T. gondii*. Finally, we showed that T. gondii-induced NETs activate neutrophils and promote the recruitment of autologous CD4^+^ T cells and the production of interferon gamma (IFN-γ), tumor necrosis factor (TNF), interleukin 6 (IL-6), IL-17, and IL-10 by peripheral blood mononuclear cells. In conclusion, we demonstrated that T. gondii activates neutrophils and promotes the release of NETs, which amplify human innate and adaptive immune responses.

## INTRODUCTION

Approximately one third of the human population is estimated to be chronically infected by the obligate intracellular parasite Toxoplasma gondii (1). Human infections occur mainly through consumption of food or water contaminated with T. gondii cysts. After establishment of infection in the small intestine, T. gondii enters the bloodstream and disseminates through different organs, such as the brain and the eye (2). T. gondii actively infects different immune cells that help its dissemination to immunoprivileged sites (3, 4).

Neutrophils are the first cells to migrate to sites of infections, with a variety of mechanisms to sense and eliminate microbes, including phagocytosis, production of reactive oxygen species (ROS), and neutrophil extracellular trap (NET) formation (5, 6). Furthermore, neutrophils have been shown to interact with T cells, amplifying the adaptive immune response (7). Previous studies in mice demonstrated that during T. gondii infection, neutrophils are rapidly recruited to the sites of infection and contribute to the production of cytokines and chemokines, such as interleukin-12 (IL-12), interferon gamma (IFN-γ), chemokine (C-C motif) ligand 3 (CCL3) and CCL4, which are protective against T. gondii infection (8, 9). In addition, exposure to tachyzoites triggers the release of NETs in mouse and human neutrophils, which limits the ability of the parasite to invade other cells and kills the tachyzoites (10).

Mice are natural intermediate hosts for T. gondii and became a very popular model to study immunological mechanisms of host resistance to microbes (11). However, since humans and mice evolved under distinct microbial pressure, their immunological mechanisms are not always the same (12). Studies evaluating the role of human neutrophils during toxoplasmosis are scarce. Hence, in the current study, we determined the activation status of human neutrophils from acutely and chronically T. gondii-infected patients and also characterized NET formation by human neutrophils that had been exposed to T. gondii. Additionally, we demonstrated the role of T. gondii-induced NET formation in the amplification of innate and adaptive immune responses.

## RESULTS

### T. gondii infection activates neutrophils and promote the formation of NETs.

**FIG 1:**
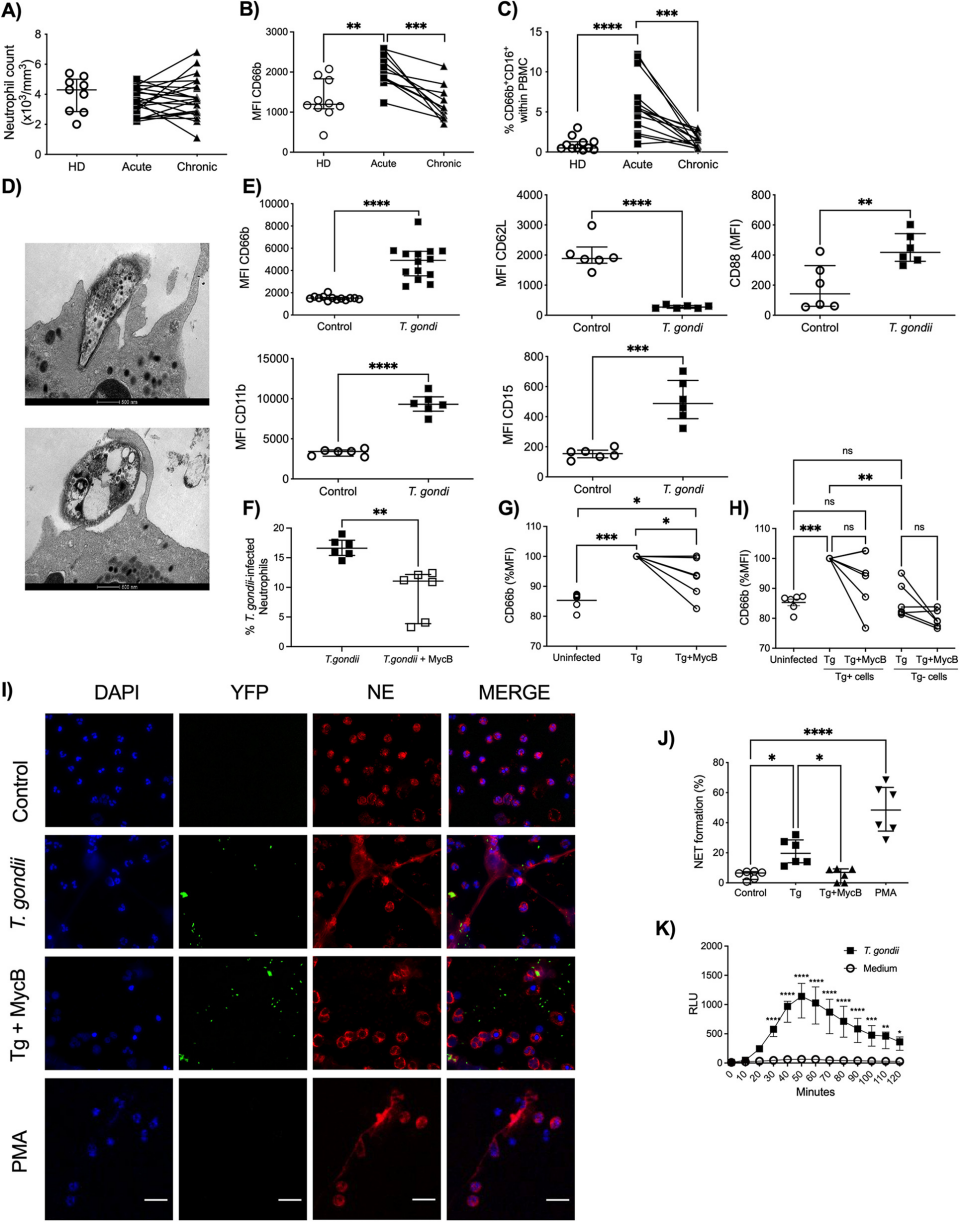
T. gondii induces neutrophil activation and NET formation. (A to C) Neutrophil absolute counts in whole blood (A), scatter dot plots of the activation marker CD66b (B), and frequency of CD66b^+^ CD16^+^ cells (C) in PBMCs from HDs (*n* = 9 to 11) and acutely (*n* = 11 to 21) and chronically (*n* = 11 to 21) T. gondii-infected patients. Each pair connected by a line represents a single individual. (D) Representative transmission electron microscopy images from a T. gondii active infection and a T. gondii opsonized infection of neutrophils from HDs. Bars, 500 nm. (E) Expression of CD66b, CD11b, CD15, CD62L, and CD88 by neutrophils from HDs in medium alone (*n* = 6 to 13) or stimulated with T. gondii tachyzoites (*n* = 6 to 14). (F) Relative expression of CD66b by neutrophils from HDs in medium alone or stimulated with T. gondii tachyzoites without (Tg) or with (Tg+MycB) mycalolide B pretreatment (*n* = 6). (G) Percentage of neutrophils infected for 4 h with T. gondii or T. gondii pretreated with mycalolide B (3 μM, 30 min) (*n* = 6). (H) Relative CD66b expression in the different subpopulations (Tg positive and negative) from panel G. (I) Representative immunofluorescence microscopy and (J) scatter dot plots (*n* = 6) of neutrophils from HDs in medium alone (Control), exposed to T. gondii RH expressing YFP with or without pretreatment with mycalolide B (3 μM, 30 min), or stimulated with 25 nM Phorbol 12-myristate 13-acetate (PMA) for 3 h. Cells were stained with Hoechst and anti-human neutrophil elastase (NE). Bars, 25 μm. (K) Neutrophil extracellular ROS production was measured by chemiluminescence (relative light units [RLU]) for 60 min in neutrophils from HDs that were unstimulated (*n* = 5) or stimulated with T. gondii (*n* = 5). The differences are relative to the control. The T. gondii-neutrophil ratio was 3:1. Data are medians and interquartile ranges. *, 0.05 > *P* > 0.01; **, 0.01 > *P* > 0.001; ***, 0.001 > *P* > 0.0001; ****, *P* < 0.0001; ns, not significant.

Neutrophils purified from peripheral blood of patients with either acute or chronic infection with T. gondii or healthy donors (HDs) were evaluated to determine their activation status. We observed that while the numbers of circulating neutrophils did not change upon T. gondii infection, during both acute and chronic phases (Fig. 1A), the frequency of expression of cell surface Cluster Differentiation 66b marker (CD66b), which is a neutrophil activation marker (13), was significantly augmented in acutely infected patients (Fig. 1B). Moreover, we assessed the presence of CD66^+^ CD16^+^ cells within peripheral blood mononuclear cells (PBMCs), which is an additional indicator of their activation. We observed a significantly increased frequency of CD66^+^ CD16^+^ cells within PBMCs from acutely infected patients, further showing that neutrophils are activated during acute toxoplasmosis (Fig. 1C).

We next examined the ability of tachyzoites of the T. gondii RH strain to activate neutrophils from HDs *in vitro*. First, using transmission electron microscopy, we observed that tachyzoites were able to both actively infect neutrophils and be phagocytosed (Fig. 1D). The expression of CD66b, CD11b, CD88, and CD15, assessed by median fluorescence intensity (MFI), was augmented while the levels of CD62L were reduced in neutrophils that were exposed to T. gondii (Fig. 1E). To determine if neutrophil activation was due to active entry or phagocytosis, we pretreated the parasites with mycalolide B, followed by multiple washes. This treatment depolymerizes the parasite’s actin, inhibiting its active entry into the cells. Flow cytometry analysis revealed that this treatment reduced the percentage of infected neutrophils from 16% to 9%, with the remaining infection presumably occurring due to phagocytosis (Fig. 1F).

We also assessed the activation status of bystander cells by the expression of CD66b in T. gondii-positive cells and T. gondii-negative cells. We found that cells that were positive for T. gondii had increased CD66b MFI, while the bystander T. gondii-negative population had CD66b levels similar to that observed in uninfected controls (Fig. 1G and H). Blocking the active entry, however, blocked the release of NETs nearly completely. This argues that NET release is not related to parasite phagocytosis and requires active entry (Fig. 1I).

The frequencies of NET formation were increased in neutrophils cocultured with tachyzoites (median, 19.6%) and neutrophils stimulated with Phorbol 12-myristate 13-acetate (PMA) (median, 48.46%) compared to nonstimulated controls (median, 6.58%). However, neutrophils incubated with T. gondii pretreated with mycalolide B showed a frequency of NET formation similar to that of nonstimulated controls (Fig. 1J). Additionally, we assessed the kinetics of ROS production during 120 min of tachyzoite exposure to neutrophils from HDs by using the luminol assay. We observed that neutrophils from HDs exposed to T. gondii induced significantly more total ROS than nonexposed neutrophils (controls). The peak of ROS production by neutrophils occurred at 50 min after T. gondii infection (Fig. 1K). Together, these results indicate that neutrophils are activated after T. gondii exposure.

### Bioenergetic profile of neutrophils exposed to T. gondii tachyzoites.

**FIG 2:**
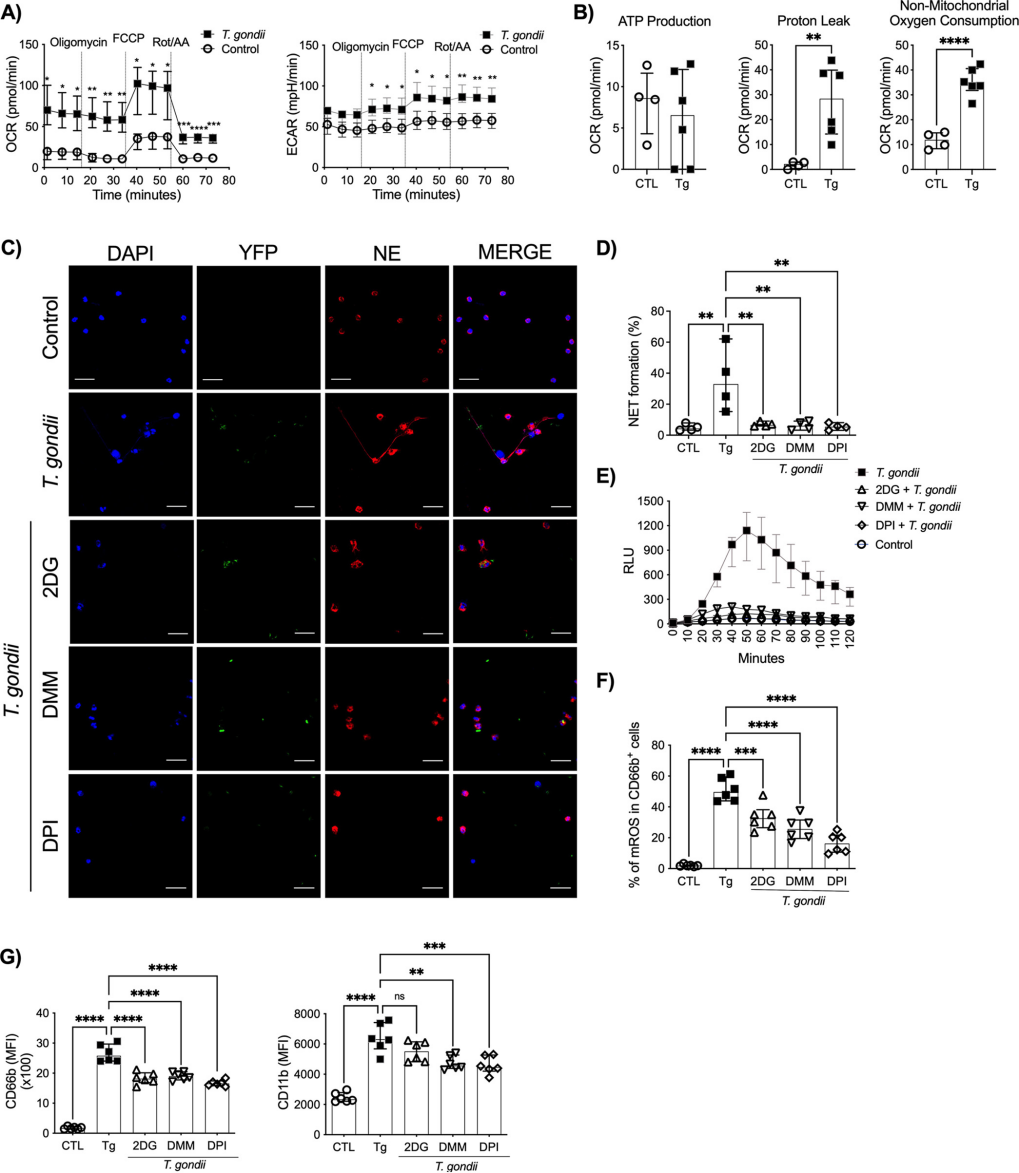
T. gondii-induced NETs are dependent on glucose and mitochondrial metabolism. OCR and ECAR (A) and ATP production, proton leakage, and nonmitochondrial oxygen consumption (B) were measured in neutrophils from HDs that were unexposed (*n* = 4 or 5) or exposed to T. gondii (*n* = 6). Representative immunofluorescence microscopy (C) and scatter dot plots (*n* = 4) (D) of neutrophils from HDs cocultured with the T. gondii YFP-expressing RH strain in the presence or absence of the metabolic inhibitors 2-DG (2 mM), DMM (10 mM), and DPI (5 μM). Cells were stained with DAPI and anti-human NE. Bars, 25 μm. (E) Kinetics of ROS production by neutrophils from HDs stimulated with T. gondii RH (T. gondii-neutrophil ratio, 3:1) or left unstimulated in the presence or absence of the inhibitors 2-DG (2 mM), DMM (10 mM), and DPI (5 μM) (*n* = 5). Luminol was measured by chemiluminescence (relative light units [RLU]) for 60 min. (F) Mitochondrial ROS were assessed by measuring MitoSox by flow cytometry in neutrophils from HDs exposed to T. gondii RH or left unexposed in the presence or absence of the metabolic inhibitors (*n* = 6). (G) MFI scatterplots of CD66b (left) and CD11b (right) in neutrophils from HDs cocultured with T. gondii tachyzoites or not cocultured in the presence or absence of the metabolic inhibitors (*n* = 6). Data are medians with interquartile ranges. *, 0.05 > *P* > 0.01; **, 0.01 > *P* > 0.001; ***, 0.001 > *P* > 0.0001; ****, *P* < 0.0001; ns, not significant.

We also characterized the bioenergetic profiles of neutrophils from HDs cocultured with T. gondii tachyzoites using Seahorse culture plates. We observed increased oxygen consumption rates (OCR) and extracellular acidification rates (ECAR) in neutrophils cocultured with tachyzoites compared to neutrophils cultured with medium alone (Fig. 2A). In addition, we assessed the ATP production, proton leakage, and the nonmitochondrial respiration. T. gondii exposure did not induce increase in ATP production by neutrophils from HDs. However, higher proton leak and nonmitochondrial oxygen consumption levels were observed in neutrophils incubated with T. gondii than in unexposed neutrophils (controls) (Fig. 2B). Furthermore, we investigated the role of glycolysis and the tricarboxylic acid cycle (TCA) in NET formation by using specific inhibitors (Fig. 2C). We observed that previous treatment of neutrophils from HDs with either 2-deoxy-d-glucose (2DG) (median, 6.57%), a glucose analog that inhibits glycolysis, or dimethyl malonate (DMM) (median, 6.03%), a succinate dehydrogenase inhibitor, diminished NET formation when neutrophils were cultured with T. gondii (median, 32.96%). As expected, pretreatment with diphenyleneiodonium chloride (DPI), a NADPH oxidase inhibitor, prevented NET formation (median, 5.46%). The frequency of NET formation in the control group (median, 4.31%) was similar to those in groups that were pretreated with 2DG, DMM, and DPI and then cocultured with tachyzoites (Fig. 2D). Interestingly, extracellular ROS (Fig. 2E) and mitochondrial ROS (Fig. 2F) production was reduced or inhibited when neutrophils from HDs were pretreated with 2DG, DMM, and DPI and then stimulated with T. gondii tachyzoites. We then confirmed that these inhibitors did not reduce parasite infectivity (Fig. S1A and B) or cell viability (Fig. S1C). Moreover, pretreatment with 2DG, DMM, and DPI reduced neutrophil activation status, as observed by diminished expression of CD66b and CD11b (MFI) compared to that in T. gondii-exposed neutrophils (Fig. 2G). In summary, these results suggest an important role of the neutrophil energetic profile in T. gondii-induced NET formation.

10.1128/mBio.01307-21.1 FIG S1 Glycolysis and mitochondrial metabolism inhibitors do not affect parasite infectivity but interfere with neutrophil activation. Representative contour plots (A) and frequency of YFP^+^ T. gondii RH in purified neutrophils (CD66b^+^ cells) from HDs (B) that were untreated or treated with 2-DG (2 mM), DMM (10 mM), and DPI (5 μM) (*n* = 6). Data are medians with interquartile ranges. **, 0.01 > *P* > 0.001; ***, 0.001 > *P* > 0.0001; ****, *P* < 0.0001. (C) Neutrophils were untreated or treated with the indicated inhibitors in the presence of T. gondii (multiplicity of infection [MOI], 3) for 3 h, and cell viability was measured by LDH release. Data are means and standard deviations, and statistical analysis was performed by one-way ANOVA with a 95% confidence interval. Download FIG S1, TIF file, 0.5 MB. Copyright © 2021 Miranda et al. 2021 Miranda et al. https://creativecommons.org/licenses/by/4.0/ This content is distributed under the terms of the Creative Commons Attribution 4.0 International license.

### Gasdermin D is essential in T. gondii-induced NETs.

**FIG 3:**
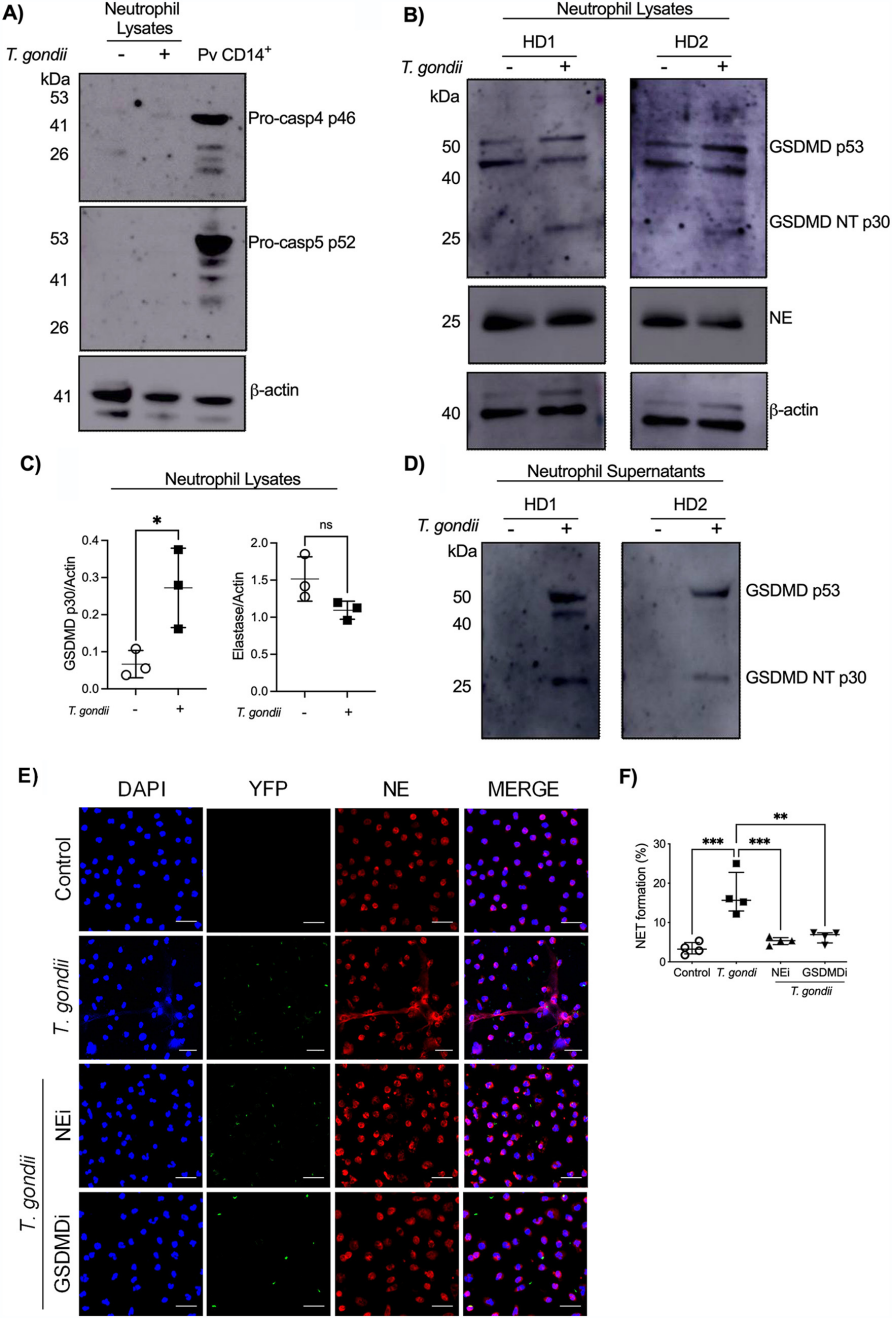
T. gondii-induced NETs are dependent on gasdermin D. Western blot analysis for caspase 4/5 (A) and for neutrophil elastase (NE) and GSDMD (B), from neutrophil lysates harvested after 3 h of T. gondii tachyzoite exposure. Pv CD14^+^, purified monocytes (CD14^+^) from Plasmodium vivax-infected patients. (C) Densitometry analysis of NE (right) and GSDMD-NT (left) bands from neutrophil lysates. Unpaired *t* test was performed. (D) Neutrophil supernatants harvested after 3 h of T. gondii exposure were precipitated for protein assessment and Western blotting performed against GSDMD. Data are representative of 2 independent experiments (A) or 3 independent experiments (B to D). (E) Representative immunofluorescence microscopy and (F) scatter dot plots (*n* = 4) of neutrophils from HDs infected with T. gondii RH expressing YFP in the presence or absence of NE and GSDMD inhibitors. Cells were stained with DAPI and anti-human NE. Bars, 25 μm. Data are medians with interquartile ranges. **, 0.01 > *P* > 0.001; ***, 0.001 > *P* > 0.0001; ns, not significant.

Next, lysates from neutrophils left unexposed or exposed to T. gondii tachyzoites were analyzed for the expression of caspase 4/5 and neutrophil elastase (NE) as potential proteases responsible for gasdermin D (GSDMD) cleavage. T. gondii tachyzoites did not induce caspase 4/5 production after 2 h of infection. As a positive control, the production of caspase 4/5 was observed in monocytes from Plasmodium vivax patients (Pv CD14^+^) (Fig. 3A). NE expression was not altered after 3 h of exposure with T. gondii (Fig. 3B). Interestingly, T. gondii tachyzoites induced increased expression of processed N-terminal GSDMD (GSDMD-NT), indicating that GSDMD is cleaved during infection (Fig. 3B). These results were confirmed by densitometry analysis of the bands from 3 independent experiments (Fig. 3C). Furthermore, we found cleaved GSDMD in neutrophil supernatants exposed to T. gondii (Fig. 3D). GSDMD expression in neutrophils has been associated with NET formation through granule membrane and plasma membrane pore formation (14). We confirmed the roles of GSDMD and NE in T. gondii-induced NETs by using GSDMD and NE inhibitors (Fig. 3E). Neutrophils previously treated with NE (median, 5.38%) and GSDMD (median, 6.92%) inhibitors and then exposed to T. gondii tachyzoites showed significantly reduced frequencies of NET formation compared to untreated neutrophils (median, 15.66%). The frequency of NET formation of nonexposed neutrophils (median, 3.23%) was similar to that of neutrophils infected with tachyzoites previously treated with NE and GSDMD inhibitors (NEi and GSDMDi) (Fig. 3F). These data indicate that T. gondii infection led to cleavage of GSDMD in a caspase 4/5-independent manner and that GSDMD is essential for T. gondii-induced NET formation.

### T. gondii-induced NETs amplify neutrophil response.

**FIG 4:**
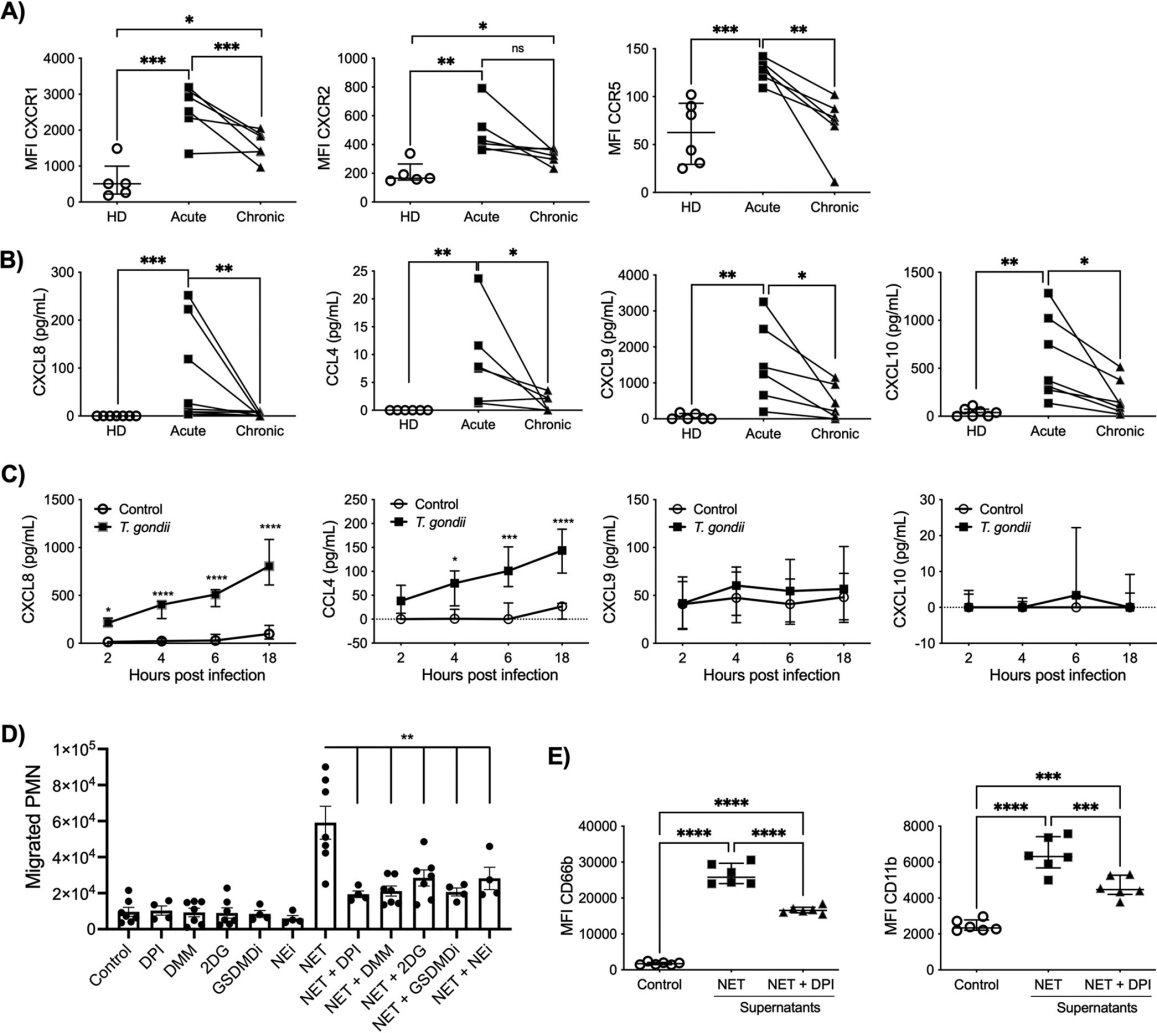
T. gondii-induced NETs amplify the neutrophil response. (A and B) Scatter dot plots of expression of the chemokine receptors CXCR1, CXCR2, and CCR5 (A) and of chemokine plasma levels of CXCL8, CCL4, CXCL9, and CXCL10 (B) in neutrophils from HDs (*n* = 5 to 7) and in neutrophils from T. gondii-infected patients during acute and (*n* = 6 to 8) chronic phase of toxoplasmosis (*n* = 6 to 8). Each pair connected by a line represents a single patient. (C) Protein levels of CXCL8, CCL4, CXCL9, and CXCL10 from neutrophils from HDs stimulated with T. gondii RH (T. gondii-neutrophil ratio, 3:1) or left unstimulated at different time points (2, 4, 6, and 18 h after infection) (*n* = 6). The differences are relative to the values obtained with the nonstimulated neutrophils. (D) Absolute numbers of neutrophils (polymorphonuclear leukocytes [PMN]) that migrated in a Transwell plate toward culture supernatants collected from neutrophils stimulated with T. gondii for 3 h (NET) in the absence or presence of 5 μM DPI, 10 mM DMM, 2 mM 2DG, 80 μM GSDMDi and 10 μM NEi and respective negative controls without T. gondii stimulation. (E) MFI in scatter dot plots of the neutrophil activation markers CD66b and CD11b on neutrophils from HDs stimulated with supernatants from T. gondii-induced NETs or left unstimulated in the absence or presence of 5 μM DPI (*n* = 6). Data are medians with interquartile ranges. *, 0.05 > *P* > 0.01; **, 0.01 > *P* > 0.001; ***, 0.001 > *P* > 0.0001; ****, *P* < 0.0001; ns, not significant.

We next evaluated expression of the chemokine receptors CXC receptor 1 (CXCR1), CXCR2, and CC receptor 5 (CCR5) in neutrophils from acutely and chronically T. gondii-infected patients as well as HDs. Acute infection induced increased levels of CXCR1 and CCR5 on neutrophils compared to HDs and chronically infected patients, while CXCR2 expression was increased in neutrophils from acutely infected patients compared to those from HDs (Fig. 4A). CXCR1 and CXCR2 promote neutrophil migration toward a CXCL8 gradient (15). We then assessed CXCL8 levels in plasma from HDs, acutely infected patients, and chronically infected patients. Similarly to CXCR1 expression, the levels of CXCL8 were significantly higher in plasma from patients with acute infection (Fig. 4B). In addition, patients with acute toxoplasmosis showed significantly higher plasma levels of CCL4, CXCL9, and CXCL10 than patients in the chronic phase of infection or HDs. Upon activation, neutrophils are known to produce a variety of chemokines, which may lead to the recruitment of different cell types (15). We then asked if neutrophils could be one of the sources of CXCL8, CCL4, CXCL9, and CXCL10 during toxoplasmosis. We evaluated the production of these chemokines in neutrophils from HDs exposed to T. gondii for 2, 4, 6, and 18 h or left unexposed. We found that after 2 h of exposure, tachyzoites induced increased levels of CXCL8 by neutrophils. Moreover, neutrophils produced significantly elevated levels of CCL4 after 4 h of T. gondii exposure. However, there were no differences in CXCL9 and CXCL10 expression levels in neutrophils from HDs exposed to T. gondii and those left unexposed (Fig. 4C).

To determine the role of T. gondii*-*induced NETs in the amplification of the neutrophil response, we collected supernatants from neutrophils exposed to T. gondii tachyzoites for 3 h (i.e., NETs) and supernatants from neutrophils pretreated with DPI, DMM, 2DG, GSDMDi, and NEi to determine their ability to inhibit NET production, in addition to their respective negative controls. These neutrophil- and parasite-free supernatants were then placed in the bottom of a Transwell plate to evaluate if they induce autologous neutrophil migration. We observed that NETs, but not NETs with inhibitors, promoted migration of neutrophils (Fig. 4D). Supernatants from neutrophils pretreated with inhibitors alone showed no alteration in migration pattern (Fig. 4D). Moreover, NET supernatants induced significantly higher levels of CD66b and CD11b on neutrophils than supernatants from neutrophils pretreated with DPI (NET+DPI supernatants) and medium alone (Fig. 4E). These results indicate that T. gondii-induced NETs promote an amplification of the neutrophil response.

### T. gondii-induced NETs amplify adaptive immune response.

**FIG 5:**
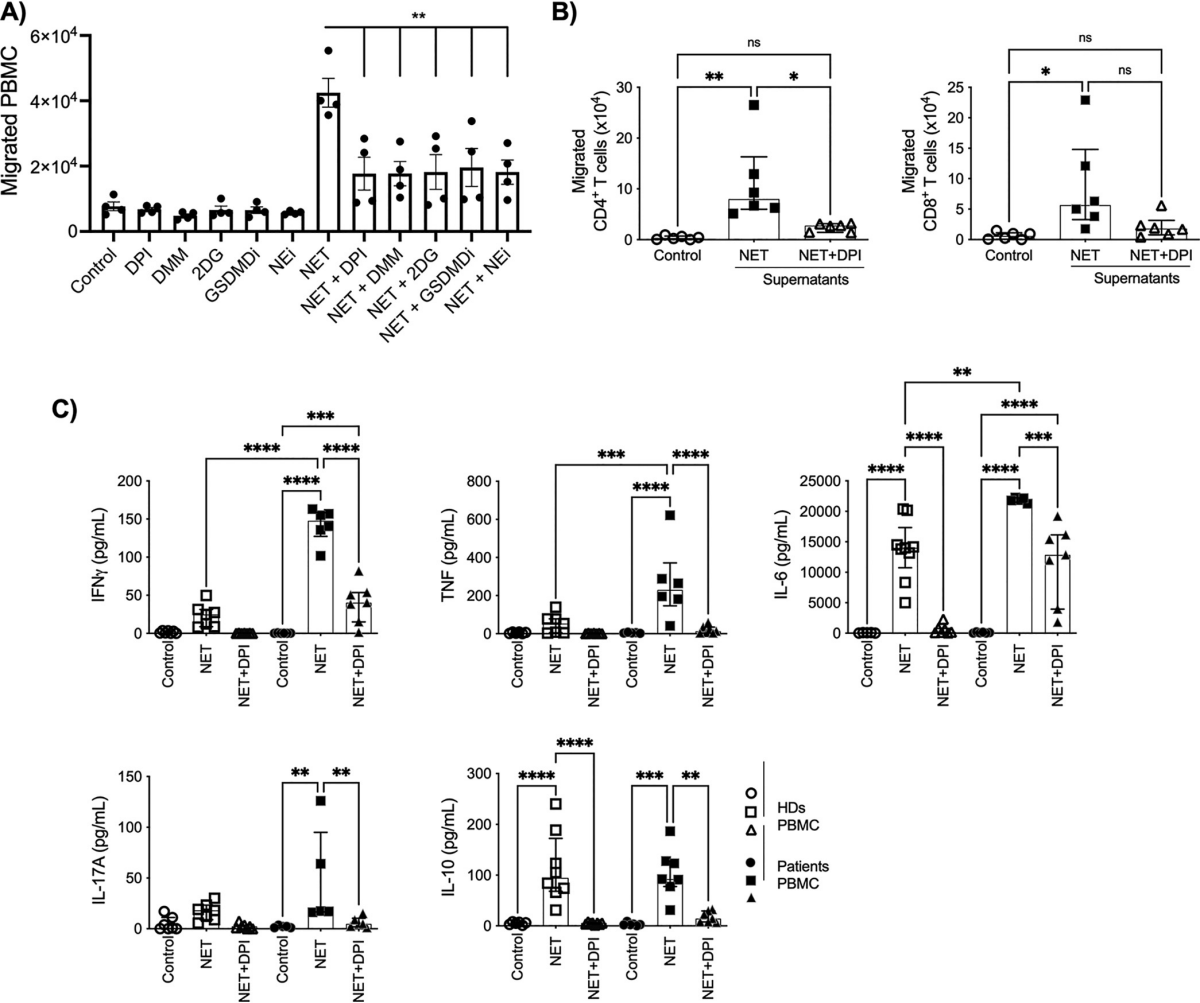
T. gondii-induced NETs amplify adaptive immune responses. (A) Absolute numbers of PBMCs that migrated in a Transwell plate toward culture supernatants collected from neutrophils stimulated with T. gondii for 3 h (NETs) in the absence or presence of 5 μM DPI, 10 mM DMM, 2 mM 2DG, 80 μM GSDMDi, and 10 μM NEi and respective negative controls without T. gondii stimulation. (B) Absolute numbers of CD4^+^ and CD8^+^ T cells that migrated in a Transwell plate toward culture supernatants collected from neutrophils stimulated with T. gondii for 3 h (NETs) in the absence or presence of 5 μM DPI (*n* = 6). (C) Levels of IFN-γ, TNF, IL-6, IL-17A, and IL-10 were assessed by cytometric beads array (CBA) in supernatants from 6 days’ culture of PBMC from HDs or from chronically infected T. gondii patients left unstimulated or stimulated with supernatants containing T. gondii-induced untreated NETs or NETs treated with 5 μM DPI. Data are medians with interquartile ranges (*n* = 6). *, 0.05 > *P* > 0.01; **, 0.01 > *P* > 0.001; ***, 0.001 > *P* > 0.0001; ****, *P* < 0.0001; ns, not significant.

To determine whether T. gondii-induced NETs impact the adaptive immune response during T. gondii infection, we evaluated the capacity of PBMCs to migrate toward supernatants from neutrophils exposed to T. gondii tachyzoites for 3 h (NETs) and supernatants from neutrophils pretreated with DPI, DMM, 2DG, GSDMDi, and NEi, in addition to their respective negative controls. PBMCs migrated toward supernatants from neutrophils exposed to T. gondii, a process that was inhibited by NET inhibitors (Fig. 5A). The capacity of CD4^+^ and CD8^+^ T cells to migrate toward autologous NET and NET+DPI supernatants showed a similar pattern. Remarkably, we observed an increased number of CD4^+^ T cells that migrated toward NET supernatants compared to NET+DPI supernatants. Although we found a trend of an increased number of CD8^+^ T cells that migrated toward NET supernatants, there were no statistically significant differences compared to cells that migrated toward NET+DPI supernatants (Fig. 5B). Next, PBMCs from HDs and from patients with chronic toxoplasmosis were cultured for 6 days in the presence of autologous NET or NET+DPI supernatants. We observed that PBMCs from chronically infected individuals produced higher levels of IFN-γ, TNF, and IL-6 when exposed to autologous NET supernatants than PBMCs from HDs. Moreover, NET supernatants induced significantly higher levels of IFN-γ, TNF, IL-6, IL-17A, and IL-10 in PBMCs from chronically T. gondii-infected individuals than NET+DPI supernatants or medium alone (Fig. 5C). Together, the results suggest that T. gondii-induced NETs may impact the adaptive immune response.

## DISCUSSION

Neutrophils play important roles in response to a variety of pathogens, including parasites (16–19). They are rapidly recruited to the sites of infection and elicit different responses to eliminate and control the microbes such as degranulation, phagocytosis, and NET formation (20–22). Through release of DNA traps along with cytosolic and granule proteins, NETs capture, neutralize, and kill microorganisms, including *Toxoplasma*, *Plasmodium*, and *Leishmania* parasites (10, 23, 24). Here, we report that human neutrophils are activated during acute infection with T. gondii and after tachyzoite exposure *in vitro*, leading to an increased expression of different cell surface markers as well as ROS production and decreased expression of CD62L. Furthermore, we observed NET formation after active tachyzoite entry in neutrophils, confirming a previous study using both human and mouse neutrophils (10).

We also demonstrated that T. gondii-induced NET and ROS production in human neutrophils is highly dependent on glycolysis and TCA metabolism by using the pharmacological inhibitors 2DG and DMM, respectively. An earlier report revealed that PMA-induced NETs are mainly dependent on glucose metabolism (25). Moreover, neutrophils use the pentose phosphate pathway (PPP) to generate NADPH, a key cofactor for NADPH oxidase to produce ROS (26). ROS are usually required for NET induction, and a shift to PPP was demonstrated to be essential for NET formation (27). NET induction is mostly dependent on ROS production, and our results confirmed these findings by using the NADPH oxidase inhibitor DPI (28, 29).

Neutrophils contain few functional mitochondria, and previous studies demonstrated that they utilize mainly glycolysis to obtain their energy supply (30, 31). Our findings corroborate previous studies, as mitochondrial ATP production was not altered in neutrophils exposed to T. gondii, while we observed increased proton leak levels and mitochondrial ROS (mROS) production in neutrophils stimulated with T. gondii. Proton leak controls mROS production by decreasing its levels, whereas mROS induce proton leaks, which suggests a protective feedback loop to control excessive mROS production induced by T. gondii (32, 33).

Besides the metabolic requirements, our study demonstrated that NET induced by T. gondii was also dependent on GSDMD cleavage. GSDMD is a protein that is cleaved by caspases and forms pores in the cell membrane that result mainly in pyroptosis (34, 35). However, our immunoblot analysis indicates that neutrophils express neither caspase-4 nor caspase-5, responsible for GSDMD cleavage in human cells, and suggest instead that elastase plays an important role in this process. Remarkably, previous studies reported an essential role of GSDMD in NET formation. It has been demonstrated that activated GSDMD forms pores in the granule membranes and in the plasma membranes of neutrophils promoting NET content release (14). Consistently, we observed that T. gondii exposure induced cleavage of GSDMD and decreased expression of NE by neutrophils, suggesting that NE had been released from these cells.

Another important role of neutrophils is the communication and interaction with other cell types. Neutrophils, after stimulation with a variety of microbes, produce chemokines that recruit more neutrophils, monocytes, dendritic cells (DCs), and T cells, which amplify both the innate and adaptive immune responses (15). Human neutrophils produce CXCL8 upon exposure to Leishmania major and Leishmania infantum species, increasing the early recruitment of neutrophils to sites of infection (36, 37). In addition, a recent report demonstrated elevated plasma levels of CXCL8 during acute toxoplasmosis (38). We confirmed these findings and further demonstrated that human neutrophils release CXCL8 after exposure to T. gondii. Furthermore, we established that T. gondii-induced NETs recruit and activate autologous neutrophils, contributing to the amplification of the innate immune response.

Neutrophils are also involved in the activation or suppression of T cells through different mechanisms, including production of soluble mediators (39–42). T. gondii infection induces high levels of circulating CXCL9 and CXCL10, chemokines known to recruit T cells (38). Moreover, primary monocytes have been shown to produce elevated levels of CXCL10 after exposure to T. gondii (43). We observed that T. gondii-induced NETs promoted CD4^+^ T cell recruitment *in vitro*. Interestingly, during NETosis, enucleated cell bodies called cytoplasts could be formed, and these cytoplasts have been shown to induce Th17 expansion through DCs in severe asthma (44, 45). Additionally, IL-17 production has been detected in patients with ocular lesions due to toxoplasmosis (46, 47). Our findings indicate that NETs contribute to IL-17 production. Moreover, phorbol 12-myristate 13-acetate PMA-induced NETs have been associated with T cell activation by reducing their activation threshold (48). Additionally, blocking NET formation reduced the frequency of IFN-γ-producing T cells in a rheumatoid arthritis mouse model, suggesting a role of NETosis in modulating Th1 response (49). Interestingly we observed that T. gondii-induced NETs also induced high levels of IFN-γ, TNF, and IL-6 in PBMCs from individuals with chronic toxoplasmosis.

In conclusion, we demonstrated that T. gondii-induced NETs are dependent on the energetic profile of neutrophils as well as in the production of ROS, elastase and GSDMD cleavage. Furthermore, the tachyzoite-induced NETs promote neutrophil and CD4 T cell recruitment as well as Th1 and Th17 cytokine release and might have important implications for the outcome of human toxoplasmosis.

## MATERIALS AND METHODS

### Patients.

Heparinized whole blood was collected from acutely T. gondii-infected patients (*n* = 21) from an outbreak that occurred in Gouveia, Minas Gerais, Brazil, in 2015 (50). These 21 patients were reassessed 6 months later and were then determined to be chronically infected. Median age at primary infection was 32 years (21.5 to 38 years), and five patients (23.8%) were female (Table 1). Peripheral blood from healthy donors (HDs) was also collected for the experiments (*n* = 17). Written informed consent was obtained before enrollment of all subjects. The study was reviewed and approved by the ethical committee on human subjects from Instituto René Rachou, Fundação Oswaldo Cruz (CEP P-8/15.2).

**TABLE 1:** Demographics of patients with postnatally acquired toxoplasmosis and healthy donors enrolled in the study

Characteristic	Patients (*n* = 21)	HDs (*n* = 17)
Age (IQR)	30 (21.5 – 38)	32 (23 – 44)
Gender		
Male (%)	16 (76.2)	7 (41.2)
Female (%)	5 (23.8)	10 (58.8)

Continuous variables are expressed as median (interquartile range [IQR]) and categorical variables as absolute number (percentage).

### Parasites.

T. gondii RH strain and transgenic T. gondii RH strain expressing yellow fluorescent protein (YFP) were used for *in vitro* experimental infections. The strains were maintained in human foreskin fibroblasts (HFF) (ATCC). In selected experiments, T. gondii was pretreated with 3 μM mycalolide B (Enzo Life Sciences) for 30 min, followed by 4 washes with phosphate-buffered saline (PBS).

### Neutrophil purification.

Neutrophils from acutely and chronically infected patients and from HDs were isolated with a Ficoll-Hypaque (GE Biosciences) gradient followed by a human neutrophil enrichment kit (STEMCELL Technologies) according to the manufacturer’s instructions. Neutrophil purity was assessed by flow cytometry.

### Cellular immunophenotyping.

Purified neutrophils from HDs cocultured with T. gondii tachyzoites or not cocultured and purified neutrophils from acutely and chronically infected patients and from HDs were stained with the following antibodies in different experimental panels: FITC (fluorescein isothiocyanate)-conjugated anti-CD66b (clone G10F5; BioLegend), anti-CD16–peridinin chlorophyll protein (PerCP)–Cy5.5 (clone 3G8; BioLegend), anti-CD11b–phycoerythrin (PE) (clone ICRF44; BD), anti-CD15–PerCP Cy5.5 (clone H198; BD), anti-CD62L–allophycocyanin (APC) (clone DREG-56; BioLegend), anti-CD88 PE (clone C85-4124; BD), anti-CD64 PE-Cy7 (clone 10.1; BD), anti-CXCR1 PE (clone 8F1; BioLegend), anti-CXCR2 APC (clone 5E8; BioLegend), and anti-CCR5 APC (clone 3A9; BD). Data are shown in frequencies or median fluorescence intensity (MFI), cells were acquired on a BD LSR-Fortessa and data were analyzed using FlowJo v10 (TreeStar).

### Transmission electron microscopy.

Neutrophils were fixed in 2.5% glutaraldehyde solution and the pellets were included in phosphate buffer with 2% agarose. Cells were fixed in a mixture of 2% osmium tetroxide and 1.5% (wt/vol) potassium ferrocyanide, dehydrated in a graded series of ethanol solutions, infiltrated, and embedded in Araldite 502 (Electron Microscopy Sciences, Hatfield, PA, USA). Specimens were cut into thin sections on an ultramicrotome (Leica EM UC6) and mounted on copper grids (Ted Pella, Inc., Redding, CA, USA), and samples were stained with 2% uranyl acetate and Reynolds lead citrate. Sections were visualized and photographed on a Tecnai G2-12 Spirit Biotwin FEI transmission electron microscopy.

### ROS detection.

For luminol-based ROS measurements, 5 × 10^5^ neutrophils from HDs were cocultured with 1.5 × 10^6^ T. gondii RH strain tachyzoites or left untreated in medium for 2 h in the presence of 100 μM luminol. Where indicated, 2DG (2 mM), DMM (10 mM), and DPI (5 μM) were added to cell cultures 30 min before stimuli. Chemiluminescence kinetics was measured with a Synergy HT (Biotek) microplate reader.

Mitochondrial ROS were assessed by flow cytometry using the MitoSox red mitochondrial superoxide indicator (Invitrogen). Briefly, neutrophils from HDs were cocultured for 2 h with T. gondii strain RH (1:3 ratio) in the presence or absence of the inhibitors 2DG (2 mM), DMM (10 mM) and DPI (5 μM) or left untreated. MitoSox (10 μM) and the monoclonal antibody anti-CD66 were added after 1.5 h of incubation. Cells were acquired by flow cytometry, and data were analyzed using FlowJo v10 (TreeStar).

### Seahorse cultures.

An XFe 96 extracellular flux analyzer (Seahorse, Agilent Technologies) was used to determine the bioenergetics profile of neutrophils stimulated with T. gondii tachyzoites. In Seahorse XF96 cell culture microplates, 5 × 10^5^ purified neutrophils were seeded and cocultured with T. gondii parasites (1.5 × 10^6^). During incubation in the XF Cell Energy Mito Stress test kit, oligomycin (1 μM), carbonyl cyanide *r*-(trifluoromethoxy)phenylhydrazone (FCCP) (0.75 μm), and rotenone (0.5 μM) were added in the respective ports. Oxygen consumption rate (OCR) and extracellular acidification rate (ECAR) were assessed under basal and stress conditions.

### Neutrophil extracellular trap induction and quantification.

For NET induction, 5 × 10^5^ neutrophils were stimulated in 4-well Lab-Tek tissue culture chambers (Thermo Fisher Scientific) with 1.5 × 10^6^ T. gondii RH organisms expressing YFP. Where indicated, DPI (5 μM), DMM (10 mM), 2DG (2 mM), NEi (10 μM), and GSDMDi (80 μM) were added to cell cultures 30 min before stimuli. As a control, neutrophils were stimulated with 25 nM PMA for 3 h. Neutrophil-free supernatants containing NETs were harvested for further use. To assess the effects of NETs in cell migration and activation, neutrophils were pretreated when indicated with 5 μM DPI to inhibit NET formation.

Neutrophils were stained with anti-human neutrophil elastase (dilution, 1:500; Calbiochem) followed by incubation with the secondary antibody anti-rabbit IgG Alexa Fluor 555 (dilution, 1:2,000; Invitrogen). Hoechst or DAPI (4′,6-diamidino-2-phenylindole) was used to counterstain the nuclei of the cells that were further mounted with Prolong antifade (Invitrogen). Images were acquired using a C2 Eclipse Ti confocal microscope (Nikon). Color images were acquired by consecutive scanning, with only one laser line active per scan to avoid cross-excitation. Data were analyzed with ImageJ software, and NET quantification was performed as described previously (51).

### Western blotting.

Neutrophils were seeded at 4 × 10^6^ per well in 6-well plates in RPMI 1640 and cocultured with T. gondii RH tachyzoites (1:3 ratio) for 3 h. Proteins from the supernatants were precipitated using an equal volume of methanol and a 1/4 volume of chloroform, and neutrophils were lysed with radioimmunoprecipitation assay (RIPA) buffer (Sigma; R0278) and a protease inhibitor cocktail (Roche; 05056489001). The lysates and precipitated supernatants were analyzed by immunoblotting using monoclonal antibodies against caspase 4 (1:1,000; Cell Signaling, 4450S), caspase 5 (1:500; Cell Signaling, 46680S), elastase (1:1,000; Abcam, ab68672), gasdermin D (1:1,000; Sigma, G7422), and actin (1:3,000; Sigma, A2066). An enhanced chemiluminescence (ECL)-based detection system was used to visualize the blots. Densitometry analysis was performed using the software Image Studio Lite V5.2.

### Chemotaxis assay.

Purified neutrophils and PBMCs (7.5 × 10^5^) from HDs were allowed to migrate for 1 h through a 5-μm-pore 96-well microchamber (NeuroProbe, Gaithersburg, MD) toward autologous NET supernatants, supernatants containing NETs combined with inhibitors (DPI, 2DG, DMM, GSDMDi, or NEi), or medium alone. Migrating cells were counted under a light microscope, and the frequencies of migrating CD66b^+^, CD4^+^, and CD8^+^ cells were evaluated by flow cytometry using specific antibodies.

### Cytokine and chemokine assay.

The levels of CXCL8, CCL4, CXCL9, and CXCL10 were measured in plasma and in culture supernatants of HD neutrophils cocultured with T. gondii RH tachyzoites (1:3 ratio) at different time points (2, 4, 6, and 18 h) by a commercially available enzyme-linked immunosorbent assay (ELISA) (BD). The levels of IFN-γ, TNF, IL-6, IL-17, and IL-10 were measured with a cytometric bead array kit for human Th1, Th2, and Th17 cytokines (BD) in culture supernatants of PBMCs from HDs and chronically T. gondii-infected individuals stimulated with autologous NET and NET+DPI supernatants or left unstimulated and in nonstimulated controls.

### LDH release assay.

A total of 2 × 10^5^ neutrophils were plated on a 96-well plate and cultured with the inhibitors DPI (5 μM), DMM (10 mM), and 2DG (2 mM) for 30 min prior to incubation with T. gondii RH tachyzoites for 3 h. The culture supernatant was assessed for lactate dehydrogenase (LDH) release using a commercial kit following the manufacturer’s instructions (CytoTox 96 nonradioactive cytotoxicity assay; Promega, G1780).

### Statistical analysis.

Results were analyzed using two-tailed paired *t* test and one-way analysis of variance (ANOVA) between groups. A Mann-Whitney or Kruskal-Wallis test was used when data did not fit a Gaussian distribution. Data shown are representative of at least two independent experiments. Differences were considered statistically significant when *P* was ≤0.05.
