# Supplementary figures and images for: Toxoplasma gondii-Induced Neutrophil Extracellular Traps Amplify the Innate and Adaptive Response
Source: mBio. 2021 Oct 5;12(5):e01307-21. doi: 10.1128/mBio.01307-21 (PMC8546543; doi:10.1128/mBio.01307-21)

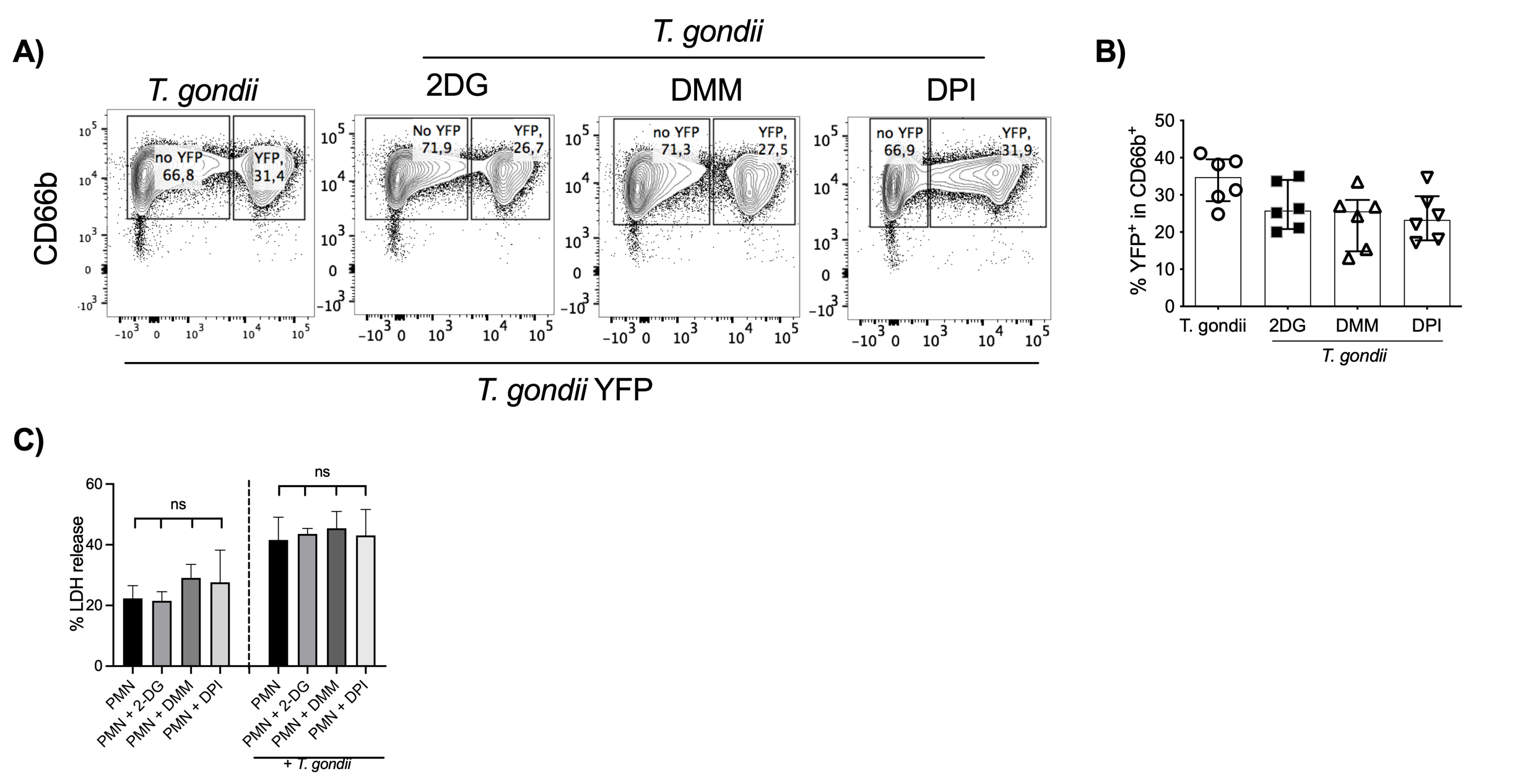

Supplement: FIG S1 [file mbio.01307-21-sf001.tif]
